# Suppression of MMP-9 by doxycycline in brain arteriovenous malformations

**DOI:** 10.1186/1471-2377-5-1

**Published:** 2005-01-24

**Authors:** Tomoki Hashimoto, Melissa M Matsumoto, Jenny F Li, Michael T Lawton, William L Young, the University of California, San Francisco, BAVM Study Group

**Affiliations:** 1 Department of Anesthesia and Perioperative Care, University of California, San Francisco, San Francisco, San Francisco, California, USA; 2 Department of Neurological Surgery, University of California, San Francisco, San Francisco, San Francisco, California, USA; 3 Department of Neurology, University of California, San Francisco, San Francisco, San Francisco, California, USA; 4 Center for Cerebrovascular Research, University of California, San Francisco, San Francisco, San Francisco, California, USA

## Abstract

**Background:**

The primary aim of this study is to demonstrate the feasibility of utilizing doxycycline to suppress matrix metalloproteinase-9 (MMP-9) in brain arteriovenous malformations (AVMs).

**Methods:**

**Ex-vivo treatment of AVM tissues: **Intact AVM tissues were treated with doxycycline for 48 hours. Active and total MMP-9 in the medium were measured. **Pilot trial: **AVM patients received either doxycycline (100 mg) or placebo twice a day for one week prior to AVM resection. Active and total MMP-9 in BVM tissues were measured.

**Results:**

**Ex-vivo treatment of AVM tissues: **Doxycycline at 10 and 100 μg/ml significantly decreased MMP-9 levels in AVM tissues ex-vivo (total: control vs 10 vs 100 μg/ml = 100 ± 6 vs 60 ± 16 vs 61 ± 9%; active: 100 ± 8 vs 48 ± 16 vs 59 ± 10%). **Pilot trial: **10 patients received doxycycline, and 4 patients received placebo. There was a trend for both MMP-9 levels to be lower in the doxycycline group than in the placebo group (total: 2.18 ± 1.94 vs 3.26 ± 3.58, P = .50; active: 0.48 ± 0.48 vs 0.95 ± 1.01 ng/100 μg protein, P = .25).

**Conclusions:**

A clinically relevant concentration of doxycycline decreased MMP-9 in ex-vivo AVM tissues. Furthermore, there was a trend that oral doxycycline for as short as one week resulted in a decrease in MMP-9 in AVM tissues. Further studies are warranted to justify a clinical trial to test effects of doxycycline on MMP-9 expression in AVM tissues.

## Background

Brain arteriovenous malformations (AVM) represent a relatively infrequent but devastating source of neurological morbidity in relatively young adults [1]. Prevention of new or recurrent intracranial hemorrhage (ICH) is the primary rationale for treating AVMs. The optimal management of AVMs is not well defined, and the risk of aggressive surgical therapy can be significantly high. There is a subset of AVM patients that are considered to be inoperable due to the location and size of their lesions [2]. To date, there is no clinically available pharmacological treatment of inoperable AVMs to decrease the rate of spontaneous intracranial hemorrhage.

Matrix metalloproteinases (MMPs), a family of proteolytic enzymes, degrade extracellular matrix proteins, cell surface molecules, and other peri-cellular substances [3]. Excessive degradation of the vascular matrix by MMPs may result in the destabilization of the blood vessel that potentially leads to weakening of the vessel wall, passive dilatation, and rupture [4]. Previously, we reported increased levels of MMP-9 activity in AVMs that may result in vascular instability associated with growth and bleeding.

There is an increasing interest in utilizing MMP inhibitors in treating vascular diseases including abdominal aortic aneurysms. Doxycycline is a clinically available antibiotic agent that possesses non-specific inhibitory effects on various MMPs, and for years it has had a well-established safety record in treating infectious diseases.

The primary aim of this study is to demonstrate the feasibility of utilizing doxycycline as an MMP inhibitor to decrease MMP-9 activity in AVMs and potentially decrease the rate of spontaneous hemorrhage. This exploratory investigation supports the concept that further studies be conducted to document the ability of tetracycline and its derivatives to decrease MMP-9 levels in AVM nidal tissue. Such a demonstration could provide a firm rationale for proceeding with clinical trials to test the hypothesis that tetracycline and its derivatives are useful to decrease the rate of spontaneous hemorrhage from AVMs in otherwise untreatable patients or in those awaiting interventional treatment.

First, we demonstrate that doxycycline can decrease MMP-9 activity in cultured AVM tissues as a proof of the concept. Further, we present results from a pilot clinical study demonstrating effects of oral doxycycline treatment on MMP-9 expression in AVM tissues.

## Methods

### Ex-vivo treatment of cultured AVM tissues

After institutional review and informed consent, we obtained AVM specimens after microsurgical resection. AVM nidus was dissected away from any adjacent brain tissue in the operating room and a representative portion of nidus tissue was used for ex-vivo treatment. Ex-vivo culture of AVM tissue was performed using previously described method with modifications [5].

AVM tissues were minced into 1–2 mm fragments and vigorously washed with phosphate buffered saline (PBS). Blood cells dissociated from the tissues were removed by filtration. AVM tissue fragments were placed onto cell culture inserts and immersed in cell culture medium. During the first 24 hours, AVM tissues were incubated in DME H-21 medium containing 10% fetal bovine serum (FBS) to aid in tissue recovery. Following this recovery period, tissue debris and dissociated cells were removed, and AVM tissues were incubated in DME H-21 medium containing 1% FBS for 4 hours. The tissue fragments were equally divided into 12–24 wells. Then AVM tissues were incubated in the medium containing PBS (control) or doxycycline (1, 5, 10 and 100 μg/ml). Each group included 3–5 wells. Size of the tissues dictated a number of treatment groups in each specimen.

Medium from each well was collected after 48 hours. Active and total MMP-9 were measured using substrate zymography. Medium was mixed with SDS sample buffer (Invitrogen, Carlsbad, CA, U.S.A.) and separated under non-reducing conditions in a 10% zymogram gel (Invitrogen) containing 0.1% gelatin incorporated as a substrate. Recombinant MMP-2 and MMP-9 proteins (R&D systems) were used as positive controls. After running, the gel was incubated with renaturing buffer (Invitrogen). The gel was then incubated with developing buffer (Invitrogen) overnight at 37°C. The gel was then stained with colloidal blue stain (Invitrogen). Proteolytic bands in the zymogram gels were quantified using Image J Software (NIH).

Tissue viability was assessed by measuring the amount of LDH (lactate dehydrogenase) released in the medium according to the manufacturer's instructions (Roche, Penzburg, Germany). Some of the tissue fragments were embedded in paraffin for histological assessment.

### Pilot clinical study

Fourteen AVM patients received either 100 mg of doxycycline or placebo twice a day for one week, prior to elective AVM resection. During the AVM resection, AVM tissues were collected, and nidus tissues were frozen in liquid nitrogen. Frozen tissues were stored at -80°C until analysis. Clinical data were collected as previously described [6].

The specimens were homogenized and insoluble materials were removed by centrifugation at 3000 rpm for 5 minutes. We used total MMP-9 ELISA kit (R&D) and active MMP-9 ELISA kit (Amersham).

### Statistical analysis

Data are presented as mean ± standard deviation. The data for total MMP-9 and active MMP-9 from ex-vivo AVM tissues are presented as a relative expression with control brain samples as 100%. We used ANOVA for comparison, and statistical significance was taken at *P *< .05.

## Results

### Ex-vivo treatment of cultured AVM tissues

We collected two AVM tissues for ex-vivo treatment with doxycycline. These patients were not enrolled in the pilot clinical study.

### AVM-I

AVM tissue-I was collected from a 28 y.o. patient with a 29 mm AVM (Spetzler-Martin score 3). The patient had an AVM hemorrhage 236 days prior to the AVM resection. The patient did not receive embolization treatment or radiosurgery. AVM tissue-I was divided into 9 wells and treated with 0, 10, and 100 ng/ml of doxycycline (3 wells for each). Two doxycycline doses were selected to test (1) whether an average doxycycline concentration (10 ng/ml) achieved by standard clinical treatment has any effects on MMP-9 in AVM tissue, and (2) whether a high concentration of doxycycline (100 ng/ml) has any effects of viability of AVM tissues in vitro.

**Figure 1:**
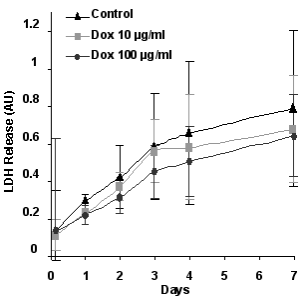
**Tissue viability assessed by LDH (lactate dehydrogenase) release. **There was a gradual increase in LDH release (mean ± SD) over the course of 8 days, indicating continuous cellular death and a gradual decrease in tissue viability. However, there was no difference among different treatment groups.

There was a gradual increase in LDH release over the course of 8 days, indicating continuous cellular death and a gradual decrease in tissue viability (Figure 1). However, there was no difference among different treatment groups. To avoid effects from ongoing cellular death and to ensure tissue viability during the treatment, we chose the first 48 hours to assess the effects of doxycycline on MMP-9 levels in AVM tissues.

**Figure 2:**
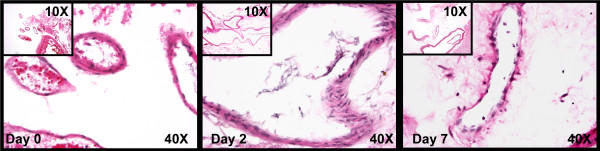
**H&E staining of ex-vivo cultured AVM tissues. **On day 0 (before the treatment), although cutting and mincing of the AVM tissues appeared to cause minor tissue injury and distortion, a majority of blood vessels was intact and viable. On day 2 (after 48 hours of treatment), blood vessels were still intact and viable, and there was no apparent necrosis or hyalinization of the tissues. However, on day 7, a major part of the tissues, especially tissues surrounding blood vessels were anuclear and hyalinized, indicating that tissues were not intact or viable anymore.

Tissue integrity was further assessed by examining H&E staining of ex-vivo cultured tissues (Figure 2). On day 0 (before the treatment), although cutting and mincing of the AVM tissues had caused minor tissue injury and distortion, a majority of blood vessels were intact and viable. On day 2 (after 48 hours of treatment), blood vessels were still intact and viable, and there was no apparent necrosis or hyalinization of the tissues. However, on day 7, a major part of the tissues, especially tissues surrounding blood vessels were anuclear and hyalinized, indicating that tissues were not intact or viable anymore.

**Figure 3:**
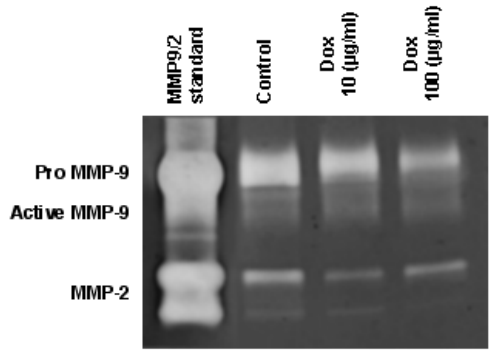
**Representative zymogram showing MMP-9 and MMP-2 standard, control AVM tissues, AVM tissues treated with 10 μg/ml doxycycline, and AVM tissues treated with 100 μg/ml doxycycline. **In AVM tissues, there were proteolytic bands corresponding to pro-MMP-9 (≈97 kDa) and active-MMP-9 (≈88 kDa).

**Figure 4:**
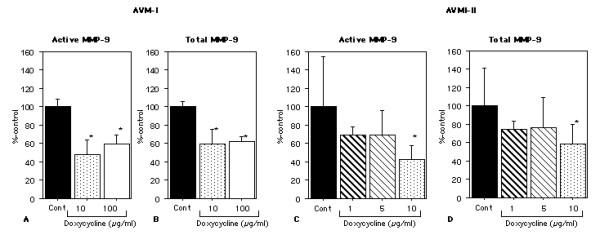
**Active and total MMP-9 levels after 48 hours of ex-vivo doxycycline treatment. AVM-I: **Doxycycline 10 μg/ml and 100 μg/ml significantly decreased active MMP-9 There was a significant reduction of total MMP-9 by doxycycline at 10 and 100 μg/ml There was no difference in active MMP-9 and total MMP-9 between doxycycline at 10 μg/ml and 100 μg/ml. **AVM-II: **Doxycycline 10 μg/ml significantly decreased active and total MMP-9. There was a trend for doxycycline 1 and 5 μg/ml to decrease active and total MMP-9. (mean ± SD)

Doxycycline 10 μg/ml and 100 μg/ml significantly decreased active MMP-9 (control vs doxycycline 10 μg/ml: 100 ± 8 vs 48 ± 16%-control, P < .05; control vs doxycycline 100 μg/ml: 100 ± 8 vs 59 ± 10%-control, P < .05) (Figure 4A). In addition, there was a significant reduction of total MMP-9 by doxycycline at 10 and 100 μg/ml (control vs doxycycline 10 μg/ml: 100 ± 6 vs 60 ± 16%-control, P < .05; control vs doxycycline 100 μg/ml: 100 ± 6 vs 61 ± 9%-control, P < .05). (Figure 4B) There was no difference in active MMP-9 and total MMP-9 between doxycycline at 10 μg/ml and 100 μg/ml. A representative zymogram is shown in Figure 3.

### AVM-II

AVM tissue-II was collected from a 63 y.o. patient with a 22 mm AVM (Spetzler-Martin score 2). The patients had a history of AVM hemorrhage 167 days prior to the AVM resection. The patient received an embolization treatment 1 day before the AVM resection. The patient did not receive radiosurgery.

Using this tissue, we aimed to study effects of doxycycline at more clinically relevant concentrations of doxycycline (1–10 μg/ml). Therefore, AVM tissue-II was treated with 0, 1, 5, and 10 μg/ml of doxycycline for 48 hours. Since the results from AVM tissue-I showed a relatively wide variation in MMP-9 levels in each well among the same treatment group, we increased wells per treatment group from 3 to 4.

There was no difference in tissue viability indicated by LDH release among different treatment groups at 48 hours (data not shown). Similar to the AVM-I, doxycycline 10 μg/ml significantly decreased active and total MMP-9 (active MMP-9: control vs doxycycline 10 μg/ml: 100 ± 54 vs 43 ± 15%-control, P < .05; total MMP-9: control vs doxycycline 10 μg/ml: 100 ± 41 vs 59 ± 21%-control, P < .05) (Figure 4C &4D). In addition, there was a trend for doxycycline 1 and 5 μg/ml to decrease active and total MMP-9.

### Pilot clinical trial

14 AVM patients were enrolled. The trial was originally started as a double blinded randomized trial, and 9 patients were randomized. In an effort to improve recruitment for this feasibility study, we converted to an open label drug design.

10 patients received doxycycline, and 4 patients received placebo. Clinical data are shown in Table 1. Subjects were informed regarding possible side effects including gastrointestinal symptoms, cutaneous photosensitivity, skin pigmentation, teeth discoloration, and vestibular side effects. To monitor compliance and possible side effects, study subjects were interviewed by phone three times during one-week treatment period. One patient had nausea that was relieved by taking food with the drug. Other side effects were not noted.

**Table 1:** Characteristics of AVM patients enrolled in a pilot clinical study to test effects of oral doxycycline treatment on MMP-9 in AVM lesions.

Group	Age	Gender	S-M score*	AVM size**	Draining veins	History of hemorrhage	Number of embolization treatments
Dox	50	M	4	38	Superficial & Deep	No	1
Dox	48	F	4	30	Deep	Yes	0
							
Dox	52	M	4	42	Superficial & Deep	Yes	2
							
Dox	43	M	3	29	Superficial & Deep	No	1
							
Dox	23	M	4	34	Superficial & Deep	No	2
Dox	28	M	4	40	Superficial	Yes	2
Dox	56	M	2	10	Superficial	No	
Dox	41	F	3	30	Superficial	No	1
Dox	62	M	2	41	Superficial	No	1
Dox	42	M	2	2	Superficial	Yes	0
							
Placebo	53	M	2	28	Superficial & Deep	No	1
Placebo	17	M	1	18	Superficial	No	1
Placebo	48	F	2	13	Deep	No	0
Placebo	22	M	3	19	Deep	Yes	0

* Spetzler-Martin score (15), **AVM maximum diameter (10). Dox = doxycycline

**Figure 5:**
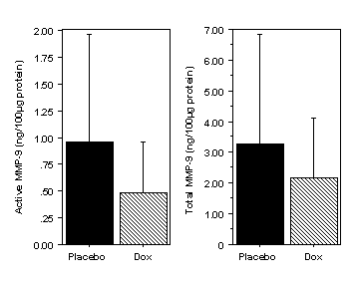
**Effects of oral doxycycline treatment on MMP-9 in AVM tissues. **There was a trend for both total MMP-9 and active MMP-9 levels to be lower in the doxycycline group than in the placebo group. (mean ± SD)

There was a trend for both total MMP-9 and active MMP-9 levels to be lower in the doxycycline group than in the placebo group (total MMP-9: 2.18 ± 1.94 vs 3.26 ± 3.58 ng/100 μg protein, P = .50; active MMP-9: 0.48 ± 0.48 vs 0.95 ± 1.01 ng/100 μg protein, P = .25) (Figure 5).

## Discussion

In this study, we demonstrated the feasibility of doxycycline, a tetracycline derivative, to decrease MMP-9 activity in AVM tissues. First, we demonstrated that a clinically relevant concentration of doxycycline decreased MMP-9 without affecting tissue viability in ex-vivo AVM tissues. Second, there was a trend that oral administration of doxycycline for as short as one week in AVM patients resulted in a decrease in MMP-9 at the target site – in the AVM nidus. By decreasing MMP-9 activity in AVM tissues, doxycycline may be able to restore the structural stability of AVM blood vessels and modify the clinical course of AVMs. Our data will provide the basis to conduct a clinical study to assess effects of tetracycline derivative treatment on the prevention of hemorrhage from AVMs.

MMP-9 is a major enzyme that degrades the vascular extracellular matrix and has been implicated in a number of vascular diseases that involve abnormal angiogenesis and vascular remodeling [7]. MMPs are reported to be increased in cerebral aneurysms, atherosclerotic carotid plaque, and abdominal aortic aneurysm [8-10]. MMPs are emerging as a potentially new therapeutic target to treat vascular diseases. It has been proposed that pharmacological inhibition of MMPs may stabilize the unstable blood vessels and prevent complications such as vessel rupture [7]. In patients with abdominal aortic aneurysm, doxycycline treatment for one week prior to the repair surgery resulted in decreased MMP-9 and MMP-2 in the wall of the aneurysms [10]. Similar results have been reported in patients with atherosclerotic carotid plaques who received doxycycline for 2–8 weeks [11].

Our data using ex-vivo AVM tissues showed that abnormally high levels of MMP-9 expression in AVM tissues could be reduced by a clinically relevant concentration of doxycycline (i.e. 1–10 μg/ml). In patients with abdominal aortic aneurysm, doxycycline treatment at a conventional dose, 200 mg/day, resulted in a mean plasma concentration of doxycycline at 4.6 μg/ml with a range of 1.3 to 14.4 μg/ml in humans, and there was a corresponding reduction in plasma MMP-9 levels [12]. Similar plasma levels of doxycycline in mice successfully inhibited growth of experimental abdominal aortic aneurysms [13]. We were able to show that doxycycline at 10 μg/ml for as short as 48 hours can reduce MMP-9 expression in AVM tissues. A longer duration of treatment often used in clinical settings may result in a more pronounced reduction in MMP-9 expression in AVM tissues.

One of the limitations of the ex-vivo study was the relatively short duration of doxycycline treatment. We chose 48 hours treatment to ensure tissue viability and integrity. Although we chose the doxycycline concentrations based on plasma doxycycline levels achieved by a commonly used therapeutic regimen, it is possible that tissue levels of doxycycline might be significantly lower than those of plasma. Furthermore, functional consequences of the reduction of MMP-9 in AVMs need to be examined in animal models or clinical studies. However, at the present time, there is no animal model that approximates recurrent intracranial hemorrhage from AVMs. There are models that can mimic certain aspects of the AVM phenotype. Hyperstimulation of mouse brain with VEGF using adenoviral transduction causes an increase in capillary density, increased MMP-9 activity, and may, in the appropriate genetic background, result in small vascular malformations [14]. Doxycycline can reduce capillary density and MMP-9 activity in this model [15].

Doxycycline has been shown in other human vascular diseases to reduce MMP levels. For example, Axisa et al. treated patients undergoing carotid endarterectomy with doxycycline or placebo for 2–8 weeks. Although MMP-1, MMP-3, and MMP-9 were reported to be increased, only MMP-1 had a statistically significant reduction by doxycycline; there was a trend for MMP-9 to be decreased by 22%. Baxter et al. used a much longer treatment period, 6 months, in patients with abdominal aortic aneurysm. They reported a gradual reduction of plasma MMP-9 levels by oral doxycycline over 6 month treatment period (baseline vs 3 months vs 6 months: 119 ± 38 vs 84 ± 33 vs 66 ± 24 ng/ml) [12].

To further explore the feasibility of a clinical application of doxycycline in modifying the clinical course of AVMs, we conducted a pilot clinical trial. This trial was primarily designed to test the effects of a short-term treatment with oral doxycycline on the expression of MMP-9 in AVM tissues. Despite the small sample size and short duration of treatment used in this study, there was a clear trend for one week of oral doxycycline treatment prior to surgical resection to reduce expression of both active and total MMP-9 in AVM tissues. These results indicate the feasibility of oral treatment with tetracycline derivatives in reducing MMP-9 activity in AVM tissues.

Despite a large difference in the mean values of MMP levels in our pilot data, variance of MMP levels was high. Accordingly, a sample size of 46 in each group would be needed to demonstrate a difference in active MMP-9, assuming an alpha of 0.05 and power of 80%. However, variance may be reduced by utilizing a longer duration of treatment, as suggested by studies in other diseases [11,12]. A clinical study with a modestly larger sample size and longer treatment duration should suffice to verify effects of oral doxycycline on reducing MMP-9 activity in AVMs.

There are some further considerations that may account for our high variance that could be avoided in future studies. Surgical AVM tissues consist mainly from nidal tissues, but they may contain intervening astroglial or neuronal tissues that may not express angiogenic factors to the same extent as nidal tissue. Histological examination of each AVM tissue fragments to confirm a predominance of nidal tissues may yield more homogenous tissue and decrease variability in MMP-9 levels. Some of the homogenized samples underwent a several "thaw-freeze" processes during the preliminary phase of the experiments, which may have increased variability in MMP-9 levels. Embolization treatment may affect production and activation of MMPs. Our previous study, however, showed no association between MMP-9 and embolization treatment in 37 AVM patients [16]. Based on the experience from this pilot study, future studies can be performed in a more standardized fashion to decrease tissue-to-tissue variability.

We used different methods to measure MMP-9 in the medium collected from ex-vivo AVM tissues and homogenized AVM tissues from the pilot clinical trial. We chose zymography, a gel based method detecting enzymatic activity of MMPs, for ex-vivo AVM tissues, because the medium volume that can be used for MMP-9 assay was extremely limited, and our preliminary experiments showed higher sensitivity of zymography compared to ELISA. One of the disadvantages of using zymography is that "gel-to-gel" variability in sensitivity makes it difficult to combine the data obtained from multiple gels. On the other hand, ELISA method allows researchers to assay approximately 40 samples at a time when duplicates are used. In addition, establishing a standard curve, ELISA method can express MMP-9 expression values by the absolute unit such as μg/ml or ng/100 μg protein. Therefore, for the clinical pilot trial, in order to quantitatively compare MMP-9 expression among a large number of samples originally planned, we used ELISA to MMP-9 expression.

## Conclusions

In summary, results suggest that doxycycline may have a therapeutic potential to reduce MMP-9 activity in AVM tissues and stabilize blood vessels that are prone to rupture. Our findings may provide a basis for a larger clinical trial to study effects of tetracycline derivatives in preventing AVM hemorrhage.

## Competing interests

The author(s) declare that they have no competing interests.

## Authors' contributions

TH, a corresponding author, participated in all aspects of the study and manuscript preparation. MMM participated in study design, experiments, analysis, interpretation of data, and manuscript preparation. JFL participated in experiments, analysis and interpretation of data. MTL participated in study design, analysis, interpretation of data, and manuscript preparation. WLY participated in study design, analysis, interpretation of data, and manuscript preparation.

## Pre-publication history

The pre-publication history for this paper can be accessed here:


